# The Influence of Emotional Intelligence on Job Burnout and Job Performance: Mediating Effect of Psychological Capital

**DOI:** 10.3389/fpsyg.2019.02707

**Published:** 2019-12-10

**Authors:** Zhun Gong, Yuqi Chen, Yayu Wang

**Affiliations:** Department of Psychology, Normal College, Qingdao University, Qingdao, China

**Keywords:** psychological capital, emotional intelligence, job performance, job burnout, intermediary effect

## Abstract

How does emotional intelligence (EI) affect job performance and job burnout? Direct or indirect? What role does psychological capital play? This study surveyed 450 employees of various enterprises through questionnaires. Results are as follows: (1) Employees’ EI has a positive predictive effect on psychological capital and job performance, and it is negatively correlated with job burnout; (2) psychological capital has a negative predictive effect on job burnout and a positive predictive effect on job performance; and (3) psychological capital plays a mediating role in the relationship between EI and job burnout/performance. Results of this study may contribute to develop EI theories in organizational behavior field. As for enterprises, improving the EI of employees will help to improve their psychological capital, and high psychological capital will lead to positive job performance and less job burnout.

## Introduction

In contemporary society, the economy is developing rapidly, competition tends to grow, and organizational performance is one of the most important indicators of how to develop ever better in the face of global competition. As a profit-making organization, improving organizational performance is the most important mission of enterprises. Present-day companies want to recruit or train potential employees who are willing to go beyond their established roles to improve performance (Adams et al., 2002). However, when enhancing the organizational performance of enterprises, the workload and work pressure of employees will also be increased. Therefore, job burnout, which can easily occur in a high-pressure environment, is a common problem in enterprises or organizations. The problem of job burnout will affect the job performance and cause it to decline, leading to more serious burnout, forming a vicious circle, and ultimately affecting the organizational performance. Earlier findings documented the fact that job burnout could have many negative effects on employees, which could not only reduce job performance and job satisfaction but also increase absenteeism/separation rate, etc. (Maslach et al., 2001). In addition, the results of a study showed that “Job burnout can easily lead to apathy toward others, have a negative impact on the individual, and have a negative impact on the organization” (Sharma and Sharma, 2015). In this context, a key to the development of enterprises is reducing employees’ job burnout and improving their job performance.

Many organizations have realized that, to stand out in today’s competitive business world, they need not only academic skills but also emotional intelligence (EI) (O’Boyle et al., 2011). EI has a long process of development; Salovey and Mayer formally put forward the concept of “EI” in 1990. In 1995, the concept of EI received much attention all over the world due to the publication of Goleman’ s book *Emotional intelligence: Why it can matter more than IQ*. Since then, an important research subject has arisen in the fields of industrial and organizational psychology: the relationship between EI and job performance. Researchers have associated job performance with EI, arguing that it was not only the ability to manage one’s own feelings but also the ability to understand others within the organization (Mayer et al., 2003). According to Bandura’s theory of self-efficacy, EI can effectively regulate individual’s emotion and then promote the formation of self-efficacy. In practice, to enable employees to adapt to new work environment faster and create better job performance, the manager also begins to pay attention to the relationship between EI and work performance in recruitment and training. If a manager improves the EI of employees, they can effectively reduce their job burnout, improve job performance, and solve the problem of poor enterprise efficiency.

Although some studies have proven the effect of EI on job performance and job burnout, the related research on how EI affects job performance and job burnout has not been fully certified and is still waiting for investigation. Pradhan confirmed the hypothesis that “EI plays a regulatory role between psychological capital and organizational citizenship behavior” (Pradhan et al., 2016). Moreover, EI and psychological capital can predict job performance and job burnout. On this basis, the purpose of this paper is to explore the role of psychological capital, which played a mediating role in the relationship between EI and job burnout. This study summarizes the four structures, gives the hypothesis, and explains the research methods. Finally, it discusses the contribution to the theoretical development and the possible implications for organization by the results.

## Theory and Hypothesis

### Emotional Intelligence

Emotional intelligence is “the ability to control emotions of oneself and others, to distinguish them from each other and to apply this information to guide one’s own thinking and action” (Salovey and Mayer, 1990). Goleman, in his book in 1995, described how to help students build up EI; his study of more than 500 organizations from the Hay Group showed that more than 85% of senior leaders owe their outstanding performance to EI rather than intelligence (Goleman, 1995). Bar-On proposed the following definition: “EI is a series of non-cognitive, competent, and skills that affect the ability of individuals to successfully respond to environmental needs and pressures” (Bar-On, 1997). In 1998, Goleman presented the concept of emotional competence, defined as follows: “On the basis of EI, people have made good achievements at work and demonstrated the ability to form through acquisition;” to distinguish emotional competence from EI, he thinks that EI is essentially a potential, and emotional competence is a kind of acquired ability based on EI, which reflects people’s ability to learn and master skills and apply this intelligence to specific situations (Goleman, 1998). Cooper noted that “if IQ is the driving force in business in the 20th century, the driving force in business in the 21st century will be EI (Cooper and Sawaf, 1998).” In 2000, Bar-On further noted that EI is a social capability that affects a range of emotional capabilities and had an effect on environmental requirements. Bar-On also made a distinction between EI and social intelligence; he regards EI as an individual’s ability to manage one’s own actions, such as impulse control, and regards social intelligence as a relational skill (Bar-On and Parker, 2000). Meanwhile, Salovey and Mayer improved and enriched the view of EI as a cognitive emotion used to enhance cognitive activities, as well as the ability to solve problems and difficulties through the use of knowledge (Mayer et al., 2000). EI includes four aspects: (1) the ability to accurately perceive, assess, and express emotions; (2) the ability to promote thinking using emotion; (3) the ability to understand emotion and emotional knowledge; and (4) the ability to regulate and manage emotions.

### Influence of Emotional Intelligence on Job Burnout and Job Performance

The concept of job burnout was proposed by Freudenberger (1974), after he put forward the concept; the problem of job burnout began to be explored by a large number of scholars. This study used Maslach’ s definition of job burnout. According to Maslach and Jackson (1981), job burnout includes emotional exhaustion, depersonalization, and personal accomplishment. Among them, exhaustion is the central quality of burnout; depersonalization is an attempt to distance yourself from the service recipient by actively neglecting the qualities that make it unique. The relationship between personal accomplishment and the other two dimensions of job burnout is more complex. It is difficult to gain a sense of accomplishment when feeling exhausted or when helping people toward whom one is indifferent (Maslach et al., 2001). Much research has been conducted to explore the antecedent variable to determine the relevant factors that prevent or reduce this negative performance.

Performance can be defined in many ways, but the more precise definition came from Campbell et al. (1993), who defined performance as “the goal relevant actions of an employee.” In other words, whether the behavior of employees matches the goals of the organization and whether it can achieve the desired results of the organization. As one of the types of performance, job performance reflects how effectively a person is using influence opportunities (Chen and Schaubroeck, 2002), that is whether the work done by the employees are effective or whether they can show good talent.

Durán et al. (2004) have shown that individuals who have a clear emotional expression ability and emotional repair ability can significantly contribute to individual success. Indeed, emotion not only affects the way people think and act but also signals about judgement and information processing (Averill et al., 1994; Brief and Weiss, 2002; Loewenstein and Lerner, 2003); employees with higher EI can find suitable solutions more smoothly at work and apply emotional resources reasonably and can often quickly access social support in communication and interaction with people, thus reducing the possibility of failure and the depersonalization brought about by failure. Employees can manage emotions by adjusting their perception of the work environment and the emotional stimuli from the environment; they can accomplish what they want to achieve by strengthening, weakening, prolonging, or shortening certain emotional experiences (Wong and Law, 2002). All of these can effectively reduce employees’ sense of burnout at work. There is also a significant negative correlation between emotional evaluation and emotional exhaustion and a significant negative correlation between emotional control and failure (Chan, 2006). In addition, the study of Platsidou (2010) showed that there is a high correlation between EI and burnout. The optimization of EI is a key factor in the relief of job burnout. Likewise, according to a systematic review of EI and teacher burnout, EI is negatively correlated with teacher burnout (Mérida-López and Natalio, 2017).

Through, these results support the hypothesis of the relationship between EI and job burnout. On the basis of the existing related theory and research summarized, we derive the following hypothesis:

*H1*: Employees’ EI has a significant negative correlation with job burnout.

*H2*: Employees’ EI has a significant positive correlation with their job performance.

### Psychological Capital

In recent years, with the hot research subject of positive psychology and its application in the fields of organizational behavior and management, a new theoretical concept, psychological capital, has been put forward. Luthans defined the concept of psychological capital as a general, positive core psychological element of the individual, including the psychological state that conforms to the standard of positive organizational behavior, which is mainly composed of four dimensions. The four positive psychological abilities of self-efficacy, hope, optimism, and tenacity are the core psychological elements that transcend economic capital, social capital, and human capital. These four dimensions are measurable and developed mental states, allowing individuals to achieve more effective performance (Youssef and Luthans, 2007). Since the positive psychological state contained in psychological capital can promote the positive attitude and behavior of the individual, empirical studies have become increasingly focused on psychological capital.

### The Influence of Psychological Capital on Job Burnout and Job Performance

The interpretation of the concept of job performance refers to a set of performance activities produced by employees in the work environment. Peterson and Luthans, through the study of enterprises, found that psychological capital, self-efficacy, optimism, hope, and tenacity can have a positive impact on job performance and attitude (Peterson and Luthans, 2003). The more “hope” employees have, the higher their performance is, in general. An empirical study on psychological capital and job performance of employees in Chinese enterprises by Luthans (Luthans et al., 2005) drew the following conclusions. First, Chinese employees’ hope, optimism, and tenacity all play a positive role in predicting their job performance. Second, the overall psychological capital, which is composed of optimism, hope, and tenacity, plays a more important role in the positive prediction of job performance. Therefore, according to the above results, the psychological capital of employees has a positive predictive effect on their job performance.

The conservation of resource (COR) theory showed that people are encouraged to acquire, protect, and promote the basic principle of acquiring what they cherish—their resources (Hobfoll, 1988). From the COR theory, people are facing the threat of losing resources in three situations. When resources are threatened (e.g., lose self-esteem because of poor grades), wasted (e.g., affect academic performance because of part-time work), and the investment in resources has no income (e.g., take time to study, but there is no increase in GPA) (Alarcon et al., 2011). Research by Cozzarelli (1993) and Rini et al. (1999) supported the view that the possession of a major resource in COR theory is usually linked with the possession of other resources. It is the desire to defend and promote acquisition of these valued resources which motivates human behavior in the face of stress (Holmgreen et al., 2017). The cross-domain relevance of COR theory makes it widely used in the study of internal pressure in organizations (Grandey and Cropanzano, 1999; Brotheridge and Lee, 2002). This is a multitiered theory designed to understand individuals nested within different environments, such as the family, the community, and the culture (Hobfoll, 2001). More importantly, it has been confirmed by many empirical studies that it is an important determinant of job burnout (Hobfoll and Shirom, 2001; Shirom et al., 2013). According to the COR theory, psychological capital can be used as an individual resource to help individuals regulate their job stress, thus alleviating their job burnout. Similarly, the meta-analysis of Lee and Ashforth (1996) showed that resource-related factors can be used to resolve emotional exhaustion and cynical negative emotions, thus effectively controlling the occurrence of low job self-efficacy situations. Avey et al. (2006) studied the correlation between psychological capital and absenteeism through an empirical study of 105 high-level engineering staff. According to the results of the study, first, optimism and hope regulated and predicted voluntary and involuntary absenteeism, and the overall psychological capital (composed of the four dimensions of optimism, hope, resilience, and self-efficacy) is more effective and accurate in predicting voluntary absenteeism than hope, optimism, resilience, and self-efficacy considered separately. Second, the results showed that there is a significant negative correlation between the overall psychological capital and involuntary absenteeism. In addition, Rehman’ s study on teachers’ job burnout showed that teachers’ positive psychological capital has a significant impact on eliminating job burnout and improving job performance (Rehman et al., 2017).

Taken together, in the theoretical support of the above research results, we can assume that employees’ psychological capital will have a negative predictive effect on their job burnout and a positive predictive effect on their job performance. Thus, the following hypotheses can be claimed:

*H3*: There is a significant negative correlation between employees’ psychological capital and job burnout.

*H4*: The psychological capital of employees has a significant positive correlation with their job performance.

### The Mediating Effect of Psychological Capital in Emotional Intelligence, Job Performance, and Job Burnout

Some studies have shown that there is a significant positive correlation between EI and psychological capital; psychological capital played a mediating role in employees’ EI and resistance to change (Malik and Masood, 2015). Meanwhile, EI is significantly related to psychological capital; in addition, employees can improve their psychological capital by motivating themselves to form self-motivation and developing the ability to control their emotions in various situations (Mellão and dos Santos Mendes Mónico, 2013).

Bandura found that the mental state is an important factor of self-efficacy (Bandura, 1978). Medium-intensity emotions contribute to the formation of self-efficacy, while overintense emotion weakens self-efficacy. EI can allow individuals to effectively regulate their emotions and promote the formation of self-efficacy. In addition, some scholars have shown that positive psychological orientation can help individuals develop better EI to maintain healthy interpersonal relationships and achieve the best organizational performance (Pradhan et al., 2016). In a recent study, it was also shown that the EI of managers plays an important role in obtaining psychological capital (Sarwar et al., 2017).

Therefore, it can be speculated that people with high EI will also have higher psychological capital. Previous studies have shown that psychological capital has a full mediating effect between servant leadership and employees’ intention to stay, sales ambidexterity, and service-oriented organizational citizenship behaviors (Tsaur et al., 2019). As a result, psychological capital, as a mediating role, has been applied in a large number of studies in the field of organizational psychology. Accordingly, the following hypothesis is suggested:

*H5*: Psychological capital plays a mediating role in the relationship between EI and job burnout/performance.

## Materials and Methods

### Participants and Procedure

This study sent out questionnaires to enterprises, organizations, or employees in advance by means of questionnaire survey. The subjects were employees of a number of private enterprises, state-owned enterprises, and public institutions in Qingdao area, Shandong Province, China, including two sales private enterprises, one IT state-owned enterprise, and one junior middle school. Participants include salespeople in the enterprise, human resources directors, and middle school mathematics teachers. To test the heterogeneity of samples, we carried out one-way ANOVA. The results showed that there was no significant difference in job performance and job burnout among different occupations (*p* > 0.05). A total of 450 questionnaires were distributed, among which 347 valid questionnaires were recovered, and the recovery rate of valid questionnaires was 77%. The basic information of the questionnaire respondents is as follows: (1) the number of male participants was 208, accounting for 59.9% of the total participants, and the number of female participants was 139, accounting for 40.1% of the total participants; (2) the number of persons with a college education or below was 166, or 47.8% of the total participants, the number of participants with undergraduate education was 174, accounting for 50.1% of the total participants, and the number of participants with a master’s degree or above was 7, accounting for 2.1% of the total participants. All the investigations were approved by the Institutional Review Board of Normal College of Qingdao University, and each participant signed informed written consent.

### Measures

#### Psychological Capital

We used the Psychological Capital Questionnaires developed by Luthans et al. (2007) to measure this variable. The scale is based on the published, widely accepted, standardized scale for all four elements to be measured, and the reliability and validity of the measurement are well validated in the context of Chinese enterprises (Luthans et al., 2008). The scale consists of four dimensions: hope, toughness, optimism, and self-efficacy. We use the six-item Likert scale to allow the participants to express opinions or attitudes from “strongly disagree” to “strongly agree.” Sample items include “since I have been through many hardships before, I am able to survive the difficult times at work now;” “I’m optimistic about what’s going to happen to my job;” “in my current job, things have never been the way I wanted them to be;” and “at work, I always believe that behind the darkness is the light; there is no need to be pessimistic.” The confirmatory factor analysis (CFA) results showed that the data are in accordance with the model of four factors and a higher-order factor [χ^2^/df = 3.63, root mean square error of approximation (RMSEA) = 0.07, comparative fit index (CFI) = 0.89, normed fit index (NFI) = 0.85, Tucker–Lewis index (TLI) = 0.88]. Using a past study as a reference, we used the total average of the four dimensions as the final measurement for the variable.

#### Emotional Intelligence

We used the Emotional Intelligence Scale (EIS) developed by Wong and Law (2002). The instruction is revised to form a Chinese version of the EIS, which consists of 16 items (Law et al., 2004) and is a self-report test. Its four dimensions are as follows: evaluation and expression of one’s own emotions; evaluation and identification of the emotions of others; monitoring of one’s own emotions; and using emotional self-motivation. The scale was scored on a seven-point scale, where “1” stands for “strongly disagree.” Sample items include “when I’m in trouble, I can control my temper and solve problems rationally;” “I can control my emotions;” “when I am angry, I usually calm down in a very short time;” and “I have good control over my emotions.” The CFA results showed that the data are in accordance with the model of four factors and a higher-order factor (χ^2^/df = 2.67, RMSEA = 0.07, CFI = 0.97, NFI = 0.96).

#### Job Burnout

We used the most authoritative and most commonly used scale in the study of job burnout—the Maslach Burnout Inventory—General Survey (Maslach et al., 1986). Li Zhaoping was authorized by Professor Michael Leiter, the developer of the questionnaire in 2002, to revise the Maslach Burnout Inventory—General Survey in the environment of China. The revised scale has good reliability and validity in China. The scale consists of three dimensions, namely, emotional exhaustion, work attitude, and sense of accomplishment. The seven-point Likert scale is used for evaluation in this study, where “zero” to “six” denote from “never” to “everyday.” Sample items include “I am confident that I can do all the work effectively;” “I’ve done much valuable work;” “I was very happy when I finished something on the job;” “in my opinion, I’m good at my job;” and “I feel like I’m making a useful contribution to the company.” The results of CFA showed that the data accord with the model of three factors and one higher-order factor (χ^2^/df = 4.63, RMSEA = 0.11, CFI = 0.93, NFI = 0.92, TLI = 0.92). According to previous studies, we take the weighted mean of the three dimensions as the final measurement of the variable.

#### Job Performance

We used the scale developed by Heilman et al. (1992) to measure employees’ performance. The scale mainly evaluates job performance through self-evaluation, including “generally speaking, very talented,” “overall, effective work” and “overall, excellent performance.” A five-point Likert scale was used for evaluation, where values from “1” to “5” range from “strongly inconsistent” to “strongly consistent.”

#### Controlled Variables

There are many empirical studies to explore the impact of demographic variables on psychological capital, job burnout, job performance, and EI. The empirical study of Gnilka and Novakovic (2017). showed that women tend to score higher in tests of emotional skills than men, Salavera also explores the gender differences in self-efficacy and EI (Salavera et al., 2017), FakhrEldin’s (2017) study on EI in innovation explores the influence of age differences. In addition, the difference in educational experience is also discussed as an influencing factor in an empirical study on employees’ EI and job satisfaction (Huang et al., 2019). On this basis, this study controlled individual traits that might have an impact on employees’ psychological capital, job burnout, job performance, and EI, including their sex, age, and educational experience. In this study, except for sex, which is classified as a variable (“one” for male and “two” for female), the other two variables are continuous. Therefore, we take an exploratory approach to the three variables.

### Data Analytic Strategy

In this study, SPSS16.0 and AMOS 23.0 software were used for data analysis. The statistical analysis items included the following: (1) descriptive statistics, correlation analysis, and diversity test of demographics and research variables; (2) reliability and validity of the questionnaire were tested by reliability analysis and CFA; and (3) the asymmetric confidence interval method (bootstrapping) was used to verify the relationship between EI, psychological capital, and job burnout/performance.

## Results

### Internal Consistency Reliability Test Results of Each Questionnaire

Reliability mainly showed whether the measurement results have good internal consistency and stability, and the higher the consistency is, the better the reliability of the scale is. The reliability analysis results are shown in Table 1. The Cronbach’s alpha reliability coefficient of each scale reached a good level above 0.8, and the reliability coefficient of each dimension of each scale exceeded 0.7, which indicates an acceptable level of reliability.

**TABLE 1:** Internal consistency reliability results of each questionnaire (*n* = 347).

	**Sample**	**Number of**	**Cronbach’s alpha**		**Number of**	**Cronbach’s alpha**
**Scale**	**sizes**	**questions**	**coefficient**	**Subscales**	**questions**	**coefficient**
Psychological capital questionnaire	*n* = 347	24	0.92	Self-efficacy	6	0.845
				Hope	6	0.844
				Tenacity	6	0.573
				Optimistic	6	0.787
Job performance questionnaire	*n* = 347	3	0.92	Job performance	3	0.916
Job burnout questionnaire	*n* = 347	15	0.87	Emotional exhaustion	5	0.929
	n=439			Working attitude	4	0.902
	n=439			Sense of achievement	6	0.908
Emotional intelligence questionnaire	*n* = 347	16	0.97	Self-assessment of emotion	4	0.925
				Other people’ s emotional assessment	4	0.909
				Emotional application	4	0.898
				Emotional control	4	0.880

### Confirmatory Factor Analysis Results of Each Research Variable

The theoretical framework of this study is based on previous theories, and the assumptions put forward are based on previous research conclusions. The research tools used in this study were revised according to previous studies; therefore, this study used AMOS23.0 to carry out the CFA of the scale. Because the job performance scale in this study has no subscales, in the CFA of this study, we do not yet analyze the research variables of job performance. Referring to the relevant research on the evaluation of structural equation model by Shook et al. (2004) the results are reported.

In this study, we used the χ^2^/df, RFI, TLI, NFI, IFI, CFI, RMSEA, and SRMSR indexes as model indicators, and the fitting criteria for determining each index are as follows: χ^2^/df ≤ 7; RFI, TLI, NFI, IFI, and CFI ≥ 0.80, where the closer the value is to 1, the better is the fit; RMSE ≤ 0.1, where the closer the value is to 0, the better is the fit; SRMSR ≦ 0.05, where the closer the value is to 0, the better is the fit.

As reported in Table 2, the results are as follows: for the chi-square test of the psychological capital scale model, χ^2^/df = 2.97 < 4; for the chi-square test of the job burnout scale model, χ^2^/df = 2.49 < 5; and the χ^2^/df of the two-scale models is <6; therefore, the fitting degree of the model is good. For the chi-square test of the EIS model, χ^2^/df = 3.94 < 7, and the fitting degree is good. The TLI, NFI, IFI, and CFI index of the psychological capital scale model is above 0.8 (TCL = 0.838, RFI = 0.774, NFI = 0.815, IFI = 0.869, CFI = 0.867); RMSEA = 0.07 < 0.08, where it reaches the level of fair fitting, therefore the degree of fitting of the psychological capital scale model to the data. The TLI, IFI, CFI, and NFI indexes of the job burnout scale model were above 0.9 (TCL = 0.956, RFI = 0.928, NFI = 0.948, IFI = 0.968, CFI = 0.968), and RMSEA = 0.06 < 0.1, it reaches the level of fair fitting; thus, the job burnout scale model has a good degree of fitting to the data. Moreover, the TLI, NFI, IFI, CFI, and RFI indexes of the EIS model were all ∼0.9 (TCL = 0.911, RFI = 0.885, NFI = 0.917, IFI = 0.937, CFI = 0.936), and RMSEA = 0.09 < 0.1, which reached the mediocre fitting level; therefore, the EIS model fits the data well. In summary, the analysis concluded that the models of the three scales well fit the data, so the above three scales all have good validity.

**TABLE 2:** Confirmatory factor analysis results of the scale (*n* = 347).

**Scale**	**χ^2^**	**df**	**χ^2^/df**	**TLI**	**RFI**	**NFI**	**IFI**	**CFI**	**RMESA**
Psychological capital	729.921	246	2.97	0.838	0.774	0.815	0.869	0.867	0.075
Job burnout	216.625	87	2.49	0.956	0.928	0.948	0.968	0.968	0.066
Emotional intelligence	386.408	98	3.94	0.911	0.885	0.917	0.937	0.936	0.092

### Confirmatory Test of Discriminant Validity of Variables

For the validation of test interpretation and the establishment of construct validity, discriminant validation is required (Campbell and Fiske, 1959). In the formal sample survey, the discriminant validity of variables was tested by CFA. According to the method of Netemeyer et al., the items of EI job burnout and psychological capital were averaged to each dimension, and each dimension was regarded as the latent variable index, while the job performance was analyzed directly by item. According to the suggestions of Fornell and Larcker (1981), we further used the average variance extracted (AVE) to test the discriminant validity of variables. The AVE of each dimension was above 0.36, in which EI (AVE = 0.54), job burnout (AVE = 0.74), and job performance (AVE = 0.73) reached a good level, and psychological capital (AVE = 0.46) was acceptable. Based on the results of CFA and the above analysis, it can be proved that the variables have good discriminant validity, so the next structural equation analysis can be carried out.

As shown in Table 3, the four-factor model (model 11) (χ^2^/df = 2.31, CFI = 0.958, RMSEA = 0.061, TLI = 0.947, RFI = 0.910, SRMSR = 0.041) is better than other nested models and has a good matching index. This means that the four-factor model can better represent the measured factor structure in this study and verify the discriminant validity of the variables.

**TABLE 3:** Confirmatory factor analysis results of measurement model.

**Model**	**χ^2^**	**df**	**χ^2^/df**	**CFI**	**TLI**	**RFI**	**RMSEA**	**SRMSR**
Model 1	1, 239.112	77	16.09	0.481	0.387	0.372	0.207	0.143
Model 2	926.871	76	12.19	0.620	0.545	0.524	0.179	0.128
Model 3	840.390	76	11.05	0.659	0.591	0.568	0.169	0.130
Model 4	935.676	76	12.31	0.616	0.540	0.519	0.180	0.148
Model 5	517.897	74	6.99	0.802	0.756	0.727	0.131	0.130
Model 6	493.390	74	6.66	0.813	0.770	0.740	0.127	0.104
Model 7	486.838	74	6.57	0.816	0.773	0.743	0.126	0.082
Model 8	608.637	74	8.22	0.761	0.706	0.679	0.143	0.086
Model 9	604.619	74	8.17	0.763	0.709	0.681	0.143	0.088
Model 10	488.636	74	6.60	0.815	0.772	0.742	0.126	0.102
Model 11	164.309	71	2.31	0.958	0.947	0.910	0.061	0.041

*Model 1: psychological capital; job burnout; job performance; emotional intelligence. Model 2: psychological capital + job burnout; job performance + emotional intelligence. Model 3: psychological capital + job performance; job burnout + emotional intelligence. Model 4: psychological capital + emotional intelligence; job burnout + job performance. Model 5: psychological capital; job burnout + job performance; emotional intelligence. Model 6: job burnout; job performance + psychological capital; emotional intelligence. Model 7: job performance; psychological capital + emotional intelligence; job burnout. Model 8: job performance; psychological capital + job burnout; emotional intelligence. Model 9: job performance; job burnout + emotional intelligence; psychological capital. Model 10: job burnout; job performance + emotional intelligence; psychological capital. Model 11: psychological capital + job performance + job burnout + emotional intelligence.*

### Descriptive Statistics and Correlation Analysis of the Research Variable

Descriptive statistics of each variable are shown in Table 4. The correlation analysis showed that there is a significant positive correlation between psychological capital and EI, a significant negative correlation between psychological capital and job burnout, a significant positive correlation between psychological capital and job performance, a significant negative correlation between EI and job burnout, and a significant positive correlation between EI and job performance. The results of these analyses are consistent with the theoretical expectations.

**TABLE 4:** Descriptive statistics of main variables.

	***M***	***SD***	**Psychological capital**	**Job burnout**	**Job performance**	**Emotional intelligence**
Psychological capital	4.60	0.59				
Job burnout	1.92	1.92	–0.19^∗∗^			
Job performance	3.70	3.70	0.19^∗∗^	–0.5		
Emotional intelligence	5.36	5.36	0.49^∗∗^	–0.16^∗∗^	0.19^∗∗^	

*^∗∗^Significant correlation at 0.01 level (two-tailed).*

### The Diversity Test of Scores of Each Scale and Demographic Variables

As mentioned above, we analyzed three demographic variables from an exploratory point of view. Through the one-way ANOVA and independent t test, the variables of each scale were tested for significant differences under different levels of demographic variables to explore the influence of each demographic variable, including gender differences, age differences, and differences in educational experience.

#### Gender Differences

Independent t test was made with sex as categorical variable and psychological capital, job burnout, job performance, and EI as dependent variables (Table 5). The men’s scores in psychological capital, job performance, and EI were slightly higher than the women’s scores, and the men’s scores of job burnout were lower than the women’s scores, but the overall difference was not significant. Gender differences did not reach a significant level in all four variables.

**TABLE 5:** *t* test on gender of each scale.

					**Sig.**
**Scale**	**Gender**	***M***	***SD***	***t***	**(two-tailed)**
Psychological capital	Male	109.8	20.05	0.471	0.639
	Female	108.4	15.45		
Job burnout	Male	38.8	12.8	0.167	0.09
	Female	41.62	15.3		
Job performance	Male	11.6	2.42	3.49	0.06
	Female	10.57	2.55		
Emotional intelligence	Male	84.70	15.39	0.566	0.572
	Female	85.77	16.78		

#### Age Differences

A one-way ANOVA was made with the age difference as an independent variable and psychological capital, job burnout, job performance, and EI as dependent variables (Table 6). There are significant differences in job burnout and job performance among different age groups; age difference is not significant for psychological capital and EI. The results showed that, at different ages, the social experience of employees gradually matures, and they become more skilled at work; in addition, repeated work will lead to burnout and boredom, so there will be a trend of rising and falling in both job burnout and job performance.

**TABLE 6:** One-way ANOVA analysis of all scales in age.

**Scale**	**Age**	***M***	***SD***	***F***	**Scale**	**Age**	***M***	***SD***	***F***
Psychological capital	Under 18	118		1.549	Job burnout	Under 18	38		9.671^∗^
	18–25	102	30.7			18–25	35.2	11.5	
	25–35	111	15.5			25–35	36.7	10.3	
	35–45	111	14.9			35–45	46.6	15.7	
	45–55	102	19.0			45–55	46.5	17.9	
	55–65	113				55–65	53.0		
Job performance	Under 18	11.0		10.9^∗^	Emotional intelligence	Under 18	92.0		1.57
	18–25	11.4	2.4			18–25	88.9	14.8	
	25–35	12.3	2.2			25–35	86.7	16.1	
	35–45	10.2	2.4			35–45	84.7	16.0	
	45–55	9.7	2.8			45–55	80.1	17.3	
	55–65	11				55–65	69		

*^∗^*p* < 0.05.*

#### Educational Experience Difference

A one-way ANOVA with educational experience as an independent variable and psychological capital, job burnout, job performance, and EI as dependent variables (Table 7) showed that job burnout (*F* = 5.09, *p* < 0.01) and job performance (*F* = 8.94, *p* < 0.001) have significant differences by educational experience; there is no significant difference between psychological capital and educational experience. Similarly, there is no significant difference between EI and educational experience.

**TABLE 7:** One-way ANOVA analysis of all scales in educational experience.

**Scale**	**Educational experience**	***M***	***SD***	***F***	**Scale**	**Educational experience**	***M***	***SD***	***F***
Psychological capital	Specialties and below	16.86	3	0.84	Job burnout	Specialties and below	4.67	2.48	5.08^∗∗^
	Undergraduate course	17.24	2.6			Undergraduate course	4.48	2.10	
	Master’ s degree or above	16.88	2.1			Master’ s degree or above	3.32	0.76	
Job performance	Specialties and below	3.32	0.76	8.83^∗∗∗^	Emotional intelligence	Specialties and below	3.21	0.89	0.6
	Undergraduate course	3.71	0.78			Undergraduate course	3.17	0.72	
	Master’ s degree or above	3.46	0.72			Master’ s degree or above	3.04	0.81	

*^∗∗^*p* < 0.01; ^∗∗∗^*p* < 0.001.*

### Research Hypothesis Test

**FIGURE 1:**
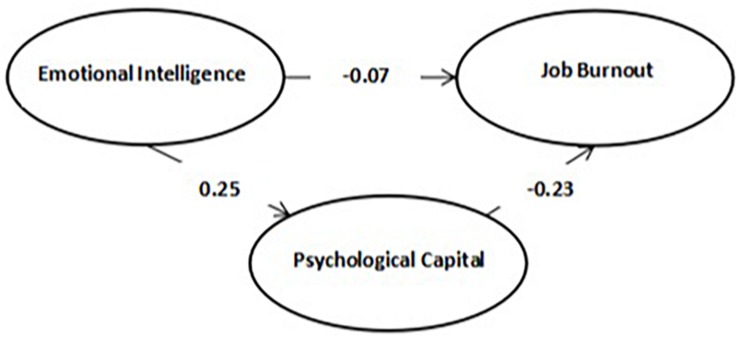
Schematic diagram of the mediating effect of psychological capital in the influence of emotional intelligence on job burnout.

To verify the theoretical hypothesis of this study, we used bias-corrected bootstrapping to examine the mediation effect. In the original data, we used the random sampling method to extract 2,000 bootstrap samples to produce approximate sampling distribution and used the 2.5% percentile and the 97.5% percentile to estimate the 95% CI of the intermediary effect. The confidence interval of the total effect does not include 0, which indicates that the mediation effect is statistically meaningful. First, we examined the role of psychological capital, which played a mediating role in the relationship between EI and job burnout (Table 8). The results showed that the confidence interval of the indirect effect is −0.133 to −0.004, which showed that a mediating role exists. The confidence interval of the direct effect is −0.182 to 0.025, which showed that psychological capital has a complete mediating role between EI and job burnout (Figure 1).

**TABLE 8:** Test the mediating effect of psychological capital in the influence of emotional intelligence on job burnout.

**Emotional**			
**intelligence–psychological**			
**capital–job burnout**	**Total effect**	**Indirect effect**	**Direct effect**
Inferior limit	–0.25	–0.133	–0.182
Superior limit	–0.039	–0.004	0.025

**FIGURE 2:**
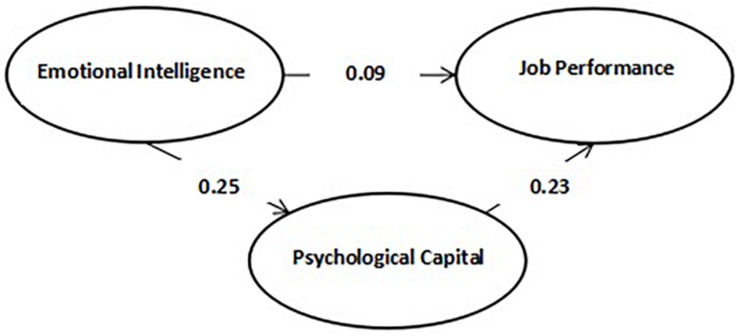
Schematic diagram of the mediating effect of psychological capital in the influence of emotional intelligence on job performance.

Second, we examined the role of psychological capital, which played a mediating role in the relationship between EI and job performance (Table 9). The results showed that the confidence interval of the indirect effect is −0.133 to −0.004, which showed that a mediating role exists. The confidence interval of the direct effect is −0.182 to 0.025, which showed that psychological capital is a complete mediating role between EI and job burnout (Figure 2).

**TABLE 9:** Test the mediating effect of psychological capital in the influence of emotional intelligence on job performance.

**Emotional**			
**intelligence–psychological**			
**capital–job burnout**	**Total effect**	**Indirect effect**	**Direct effect**
Inferior limit	0.046	0.008	−0.03
Superior limit	0.255	0.124	0.22

## Discussion

In this study, we use two frameworks shown in Figures 1, 2 to present the relationship between EI and job burnout/performance and find that psychological capital plays a mediating role in the relationship between EI and job burnout/performance. Most of previous studies focus on simple effect of EI on job performance (Wen et al., 2019), without in-depth research on mechanism behind it; therefore, this study is necessary for further exploration.

### Findings and Theoretical Contributions

First, the results of this study show that employees’ EI plays a significant positive role in predicting their job performance. In other words, in enterprises or organizations, the higher the level of EI employees have, the better they perform. It can also be speculated that employees with higher levels of EI will perform better than those with lower levels of it. Employees with high level of EI perform well and have higher satisfaction with their jobs; what is more, they also build a good social support system for their partner (Miao et al., 2017). Thus, EI plays an important role in improving enterprises’ competitive advantage. In addition, this study supports previous studies, emphasizing the correlation between EI and job burnout (Schoeps et al., 2019) and confirming that the higher employees’ EI level, the better their job performance and the lower the EI of employees, the higher their job burnout. It implies that employees’ EI levels negatively predict job burnout. The results can provide a breakthrough point for managers of enterprises or organizations to reduce employees’ job burnout. They can use different emotions to produce the best way to solve daily problems and offset dissatisfaction in work (Nǎstasǎ and Fǎrcaş, 2015). As the results prove, efforts to improve employees’ EI level will effectively slow down or eliminate employees’ job burnout. A more important finding in the results is that, adopting the role of a mediation, psychological capital can provide a theoretical basis for enterprises or organizations to improve employees’ job performance and reduce their job burnout. In fact, the experience of achievement at work will improve the psychological capital level of employees (Madrid et al., 2018). Therefore, in daily management, enterprises or organizations should pay attention to not only the job performance of employees but also the development of their psychological capital to achieve common progress of management and performance.

In sum, this study discusses the influence of EI by introducing the concept of psychological capital. It is of great innovative value to this study of organizational behavior and the construction of a healthy organization. In addition, in recent years, EI has become the leading area of academic research. In the field of business, it has also become an important part. Therefore, EI occupies the position of hot research field in the world.

Building on previous studies, this study expands the relevant theories and adds strength to the development of EI theory. The level of EI affects the ability of employees to express their personal feelings and communicate with others (Siahaan, 2018), and it will affect their handling of emotions with customer, which is related to not only the work attitude of employees but also job performance to a certain extent.

### Limitations and Future Directions

There are still some limitations to this study. First, the research variable of job performance is measured by employees’ self-report; this may entail the influence of some social expectations and tendencies in employees’ evaluation of their behavior (Podsakoff et al., 2012). However, self-report may lack objectivity, which does not conform to the real situation of employees, so there may be some deviation in the overall research. Therefore, future researchers can use the combination of self-evaluation and other evaluation to increase the objectivity of the results. Second, for sampling convenience, the samples of this study are from various enterprises or organizations in certain areas of China, which limits the generalization of the results of this study. Whether the results can be applied to other regions or industries with other cultural backgrounds needs to be further tested. To overstep this limitation of this study, future research can expand the scope of research, repeat research in different cultural areas, and can be used as a comparative study to explore the differences between the cultural background of the East and the West.

### Practical Implications

Based on these findings, we put forward some practical suggestions on the recruitment of employees and the improvement of organizational performance. First of all, when interviewing, interviewers should pay attention to the personal quality of applicants. HR managers should look for applicants who have positive attitude and genuine care (Choi et al., 2019). As for measurement method, the EIS developed by Wong and Law (2002) can be used to measure the EI of applicants in recruitment. Second, introduce regular training programs (e.g., PsyCap short training interventions) to improve employees’ EI and psychological capital (Avey et al., 2009). Third, in addition to improving the overall EI and psychological capital level of employees, the prevention of job burnout is also an important part. Enterprises need to carefully consider workload and work hours of staff and draw up appropriate work plans, which lead to well job performance and less job burnout.

Conclusively, enterprises can improve their organizational performance and development by improving the EI and psychological capital of their employees. The results of this study can be applied not only to enterprises but also to other organizations, such as schools or hospitals. Thus, this study can also provide a relevant theoretical basis for the development of multilevel and multifaceted organizations and groups.

## Conclusion

This study explores the role of psychological capital, which played a mediating role in the relationship between EI and job performance/burnout. The results showed that (1) EI is negatively correlated with job burnout, (2) EI has a significant positive correlation with job performance, (3) EI has a significant positive correlation with psychological capital, (4) psychological capital has a significant negative correlation with job burnout, (5) psychological capital has a significant positive correlation with job performance, (6) psychological capital played a mediating role in the relationship between EI and job burnout, and (7) psychological capital played a mediating role in the relationship between EI and job performance.

## Data Availability Statement

The raw data supporting the conclusions of this article will be made available by the authors, without undue reservation, to any qualified researcher.

## Ethics Statement

The studies involving human participants were reviewed and approved by the Institutional Review Board, Normal College, Qingdao University. The patients/participants provided their written informed consent to participate in this study.

## Author Contributions

ZG and YC designed, performed, and analyzed the research. ZG, YC, and YW wrote the manuscript. YW critically reviewed and edited the manuscript.

## Conflict of Interest

The authors declare that the research was conducted in the absence of any commercial or financial relationships that could be construed as a potential conflict of interest.
